# Integrated Bioinformatics Analysis Identifies Hub Genes Associated with the Pathogenesis and Prognosis of Esophageal Squamous Cell Carcinoma

**DOI:** 10.1155/2019/2615921

**Published:** 2019-12-26

**Authors:** Hui Zhang, Jianing Zhong, Youbing Tu, Benquan Liu, Zhibo Chen, Yunchen Luo, Yaping Tang, Fei Xiao, Jincai Zhong

**Affiliations:** ^1^Department of Anesthesiology, First Affiliated Hospital of Guangxi Medical University, Nanning, Guangxi, China; ^2^Department of Clinical Laboratory, First Affiliated Hospital of Guangxi Medical University, Nanning, Guangxi, China; ^3^Department of Cardiothoracic Surgery, First Affiliated Hospital of Guangxi Medical University, Nanning, Guangxi, China; ^4^Department of Endocrinology and Metabolism, Second Affiliated Hospital of Guangxi Medical University, Nanning, Guangxi, China; ^5^Department of Medical Oncology, First Affiliated Hospital of Guangxi Medical University, Nanning, Guangxi, China

## Abstract

Esophageal squamous cell carcinoma (ESCC) accounts for over 90% of all esophageal tumors. However, the molecular mechanism underlying ESCC development and prognosis remains unclear, and there are still no effective molecular biomarkers for diagnosing or predicting the clinical outcome of patients with ESCC. Here, using bioinformatics analyses, we attempted to identify potential biomarkers and therapeutic targets for ESCC. Differentially expressed genes (DEGs) between ESCC and normal esophageal tissue samples were obtained through comprehensive analysis of three publicly available gene expression profile datasets from the Gene Expression Omnibus database. The biological roles of the DEGs were identified by Gene Ontology (GO) annotation and Kyoto Encyclopedia of Genes and Genomes (KEGG) pathway analysis. Moreover, the Cytoscape 3.7.1 platform and subsidiary tools such as Molecular Complex Detection (MCODE) and CytoHubba were used to visualize the protein-protein interaction (PPI) network of the DEGs and identify hub genes. A total of 345 DEGs were identified between normal esophageal and ESCC samples, which were enriched in the KEGG pathways of the cell cycle, endocytosis, pancreatic secretion, and fatty acid metabolism. Two of the highest scoring models were selected from the PPI network using Molecular Complex Detection. Moreover, CytoHubba revealed 21 hub genes with a valuable influence on the progression of ESCC in these patients. Among these, the high expression levels of five genes—SPP1, SPARC, BGN, POSTN, and COL1A2—were associated with poor disease-free survival of ESCC patients, as indicated by survival analysis. Taken together, we identified that elevated expression of five hub genes, including SPP1, is associated with poor prognosis in ESCC patients, which may serve as potential prognostic biomarkers or therapeutic target for ESCC.

## 1. Introduction

Esophageal carcinoma (EC) ranks seventh in incidence and sixth in mortality worldwide, with approximately 572,000 new cases and 50,900 deaths due to EC estimated in 2018 alone [1]. The incidence rate of EC greatly differs depending on sex and population, with about 70% of cases occurring in men, and their mortality rate is 2-3-fold higher than that reported for women with EC. Based on the histological type, EC is classified as esophageal adenocarcinoma (EAC) and esophageal squamous cell carcinoma (ESCC), with the latter accounting for over 90% of all esophageal tumors [1]. Smoking, alcohol consumption, and their synergistic effects are the major risk factors for the development of ESCC [2, 3], whereas obesity, gastroesophageal reflux disease, and Barrett's esophagus are the key risk factors for EAC [3]. The 5-year overall survival (OS) rate for patients with ESCC remains low at 10–20% [4]. Early diagnosis, and successful surgery, radiation therapy, and chemotherapy all contribute to a better prognosis for patients with EC. However, there are still numerous challenges to achieving an early diagnosis and accurate prognosis of individual EC patients based on current clinical indicators.

In recent years, several molecular biomarkers with potential value in predicting the development of EC have been screened through high-throughput techniques, which can also help to reveal the molecular characteristics of cancer cells to predict the prognosis of patients. Based on a genome-wide association study of patients with ESCC, five genes showing relatively higher expression levels than those in controls (TDG, MBL2, CASP8, PLCE1, and UCP3) were estimated to be closely associated with an increased risk of tumor development [5]. Moreover, another study suggested that glutathione peroxidase 7 plays an important physiological role in protecting the healthy esophageal epithelium from acidic bile salt-induced oxidative stress, oxidative DNA damage, and double-stranded DNA breaks [6]. Despite these clues, the molecular mechanisms underlying ESCC development remain unclear, and there is a lack of effective molecular biomarkers to diagnose and predict the prognosis of ESCC.

**Figure 1:**
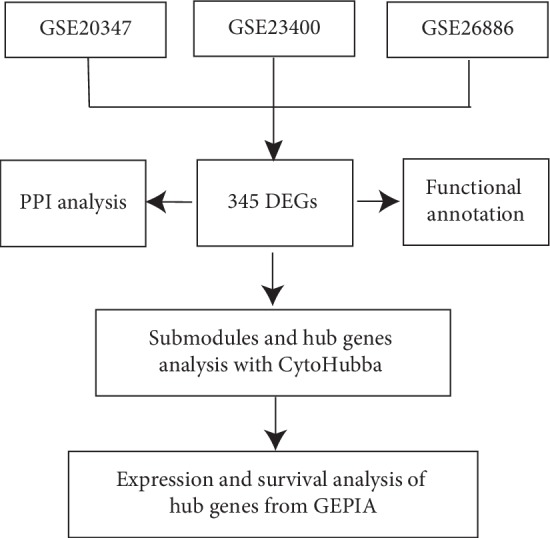
Workflow to identify molecular signature markers associated with esophageal squamous cell carcinoma (ESCC) from the GEO database.

Therefore, to identify candidate biomarkers for ESCC, we used a bioinformatics approach to analyze publicly available microarray data (GSE20347, GSE23400, and GSE26886) from the Gene Expression Omnibus (GEO) (http://www.ncbi.nlm.nih.gov/geo/) database [7]. We first determined the differentially expressed genes (DEGs) between tumor and normal esophageal tissues through an integrated analysis of the datasets. The main biological functions of the identified DEGs were then explored by Gene Ontology (GO) annotation and Kyoto Encyclopedia of Genes and Genomes (KEGG) pathways analysis. In addition, the protein-protein interaction (PPI) network of DEGs was used to identify the hub genes, and genes with a strong influence on the pathogenesis and prognosis of ESCC patients were selected through survival analysis. The workflow of the analysis is schematically shown in Figure 1.

## 2. Material and Methods

### 2.1. Data Collection

Gene expression profiles (GSE20347, GSE23400, and GSE26886) of cancerous and healthy esophageal tissues were downloaded from the GEO database [8–10]. The detailed information of the datasets is provided in Table 1. The tumor samples were isolated from ESCC patients during surgery, and none of the patients had received prior treatment before surgery. Healthy esophageal tissues were collected from patients experiencing esophageal pain but without esophageal pathological changes, or from the normal adjacent tissues of ESCC patients paired with the tumor samples.

### 2.2. Data Processing and Identification of DEGs

The raw data of the mRNA expression profiles were analyzed by the oligo package from Bioconductor (http://www.bioconductor.org/) in *R* language software [11]. The limma [12] package was then applied to select the DEGs between the ESCC and healthy samples according to the cut-off criteria of a |log 2 fold change (FC)| ≥1 and adjusted *P* value <0.05. Overlapping DEGs among the three datasets were determined with the venn diagrams packages [13] and retained for subsequent analyses.

### 2.3. Functional Enrichment Analysis of DEGs

To clarify the probable biological processes (BP), cellular components (CC), and molecular functions (MF) correlated with the common DEGs in the three datasets, GO annotation and KEGG pathway enrichment analyses were carried out by ClusterProfiler [14]. An adjusted *P* value <0.05 was considered statistically significant.

### 2.4. PPI Network, Submodules, and Hub Genes Analysis

The potential interactions of the overlapping DEGs were analyzed using the STRING [15] database, which collects and integrates information of functional interactions between expressed proteins. The network with a confidence score ≥0.4 in STRING was retained and then input to Cytoscape (version 3.7.1) [16] for visualization. In addition, we performed module analysis to detect hub clustering modules in the network utilizing the MCODE [17] application with default parameters. The significant modules were then subject to GO annotation and KEGG pathway enrichment analyses for functional interpretation. The top 20 genes were selected according to 12 different analysis methods in the CytoHubba application, which provides a user-friendly interface to analyze the topology of PPI networks [18]. Genes detected with at least five different methods were considered as the hub genes.

### 2.5. Expression and Survival Analysis of Hub Genes

GEPIA (http://gepia.cancer-pku.cn/) [19] is a newly developed interactive web server for analyzing the RNA-sequence expression data of 9,736 tumors and 8,587 normal samples from TCGA and GTEx projects. GEPIA also provides the option of conducting OS or disease-free survival (DFS) analysis based on relative gene expression levels by the log-rank test and Mantel-Cox test. Moreover, the Cox proportional hazard ratio (HR) and the 95% confidence interval (95% CI) of the survival plot can be obtained.

## 3. Results

### 3.1. Identification of DEGs

**Figure 2:**
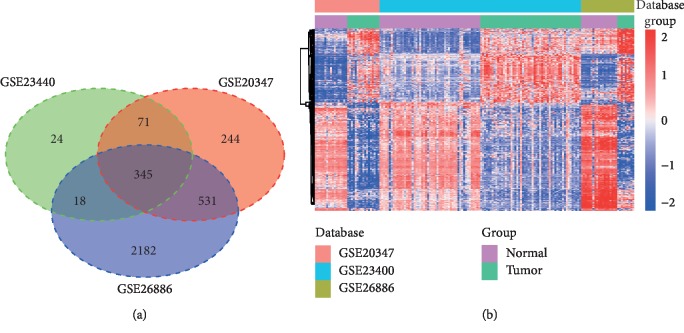
Identification of differentially expressed genes (DEGs) in ESCC. (a) A total of 345 overlapping genes were identified from the three datasets with the venn diagrams package. (b) Heat map of overlapping DEGs in ESCC and normal esophageal tissues; each column represents one sample and each row represents one gene.

Through integrated analysis of the three datasets (Table 1), a total of 345 overlapping DEGs (including 142 upregulated and 203 downregulated DEGs) were screened based on the cut-off criteria of |log 2FC| ≥1 and adjusted *P* value <0.05 (Figure 2(a) and [Supplementary-material supplementary-material-1]).  Figure 2(b) displays the heat map of overlapping DEGs among the three datasets.

### 3.2. Functional Enrichment Analysis of DEGs

**Figure 3:**
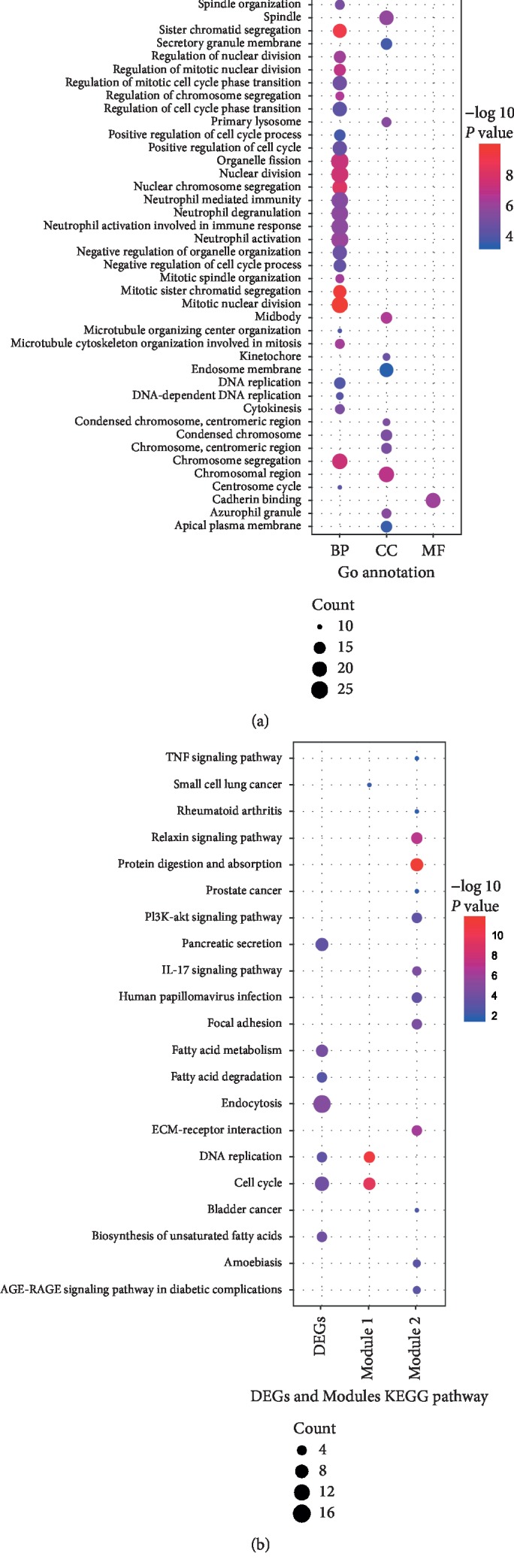
GO and KEGG pathway analyses of DEGs in ESCC. (a) BP, CC, and MF of the DEGs with GO annotation. (b) KEGG pathway enrichment analysis of the DEGs and the two modules.

GO annotation showed that the 345 DEGs were mostly enriched in the chromosome region, azurophilic granule, endosome membrane, and secretory granule membrane for the CC terms, and in cell cycle-related BP terms such as chromosome segregation and nuclear division, and neutrophil-related BP terms such as neutrophil-mediated immunity, neutrophil degranulation, neutrophil activation, and neutrophil activation involved in immune response (Figure 3(a)). Similarly, KEGG pathway enrichment analysis showed that the DEGs mainly participated in endocytosis, cycle cell, pancreatic secretion, and fatty acid metabolism pathways (Figure 3(b)).

### 3.3. PPI Network, Submodules, and Hub Genes

**Figure 4:**
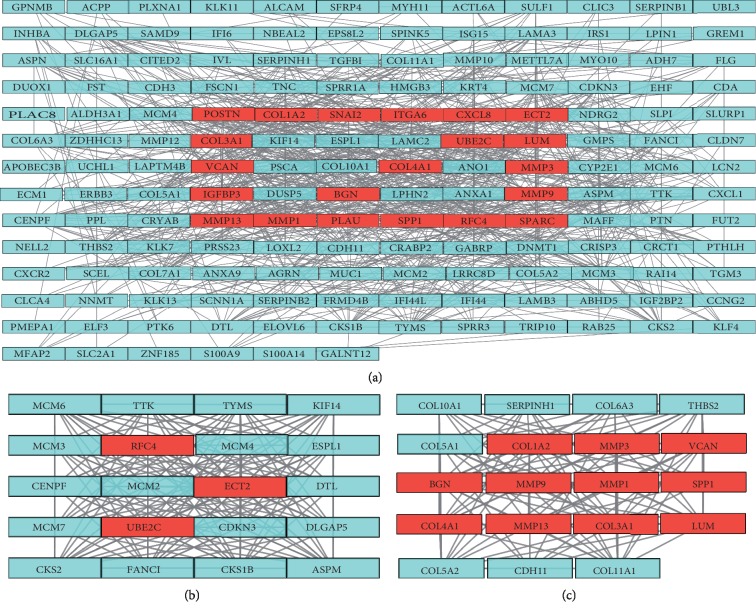
Protein-protein interaction (PPI) network and hub clustering modules. (a) The PPI network of overlapping DEGs. (b) Module 1 (MCODE score = 19.368). (c) Module 2 (MCODE score = 15.566). Red rectangles represent hub genes.

A total of 162 nodes and 778 interactions of the overlapping DEGs were identified in the PPI network, which were visualized in Cytoscape (Figure 4(a)). The CytoHubba application identified 71 hub genes, including 21 genes that were identified by at least five different methods as the candidate hub genes (Table 2). In addition, the top two clustering modules (scores: 19.368 and 15.556) were obtained with the MCODE application (Figures 4(b) and 4(c)), and the genes involved in the modules were functionally annotated. Pathway enrichment analysis indicated that these two modules were mainly correlated with DNA replication, cell cycle, protein digestion and absorption, relaxin signaling pathway, ECM-receptor interaction, IL-17 signaling pathway, and focal adhesion (Figure 3(b)).

### 3.4. Expression and Survival Analysis of Hub Genes

**Figure 5:**
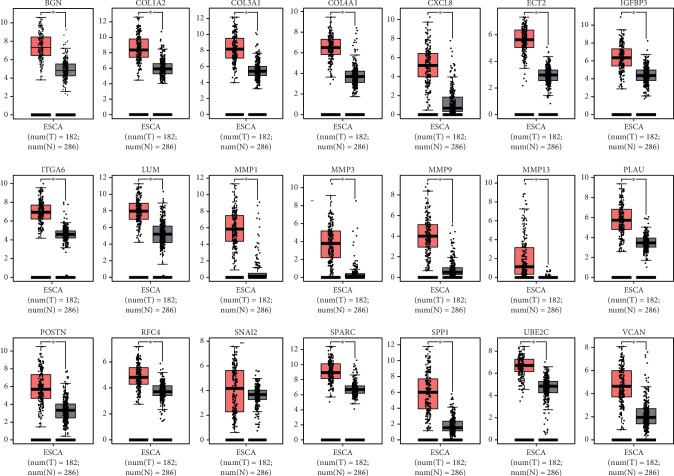
Expression of hub genes in ESCC and normal tissues from GEPIA (http://gepia.cancer-pku.cn/index.html). The expression levels of all hub genes in cancer patients were significantly higher than those in healthy controls, except for SNAI2.

**Figure 6:**
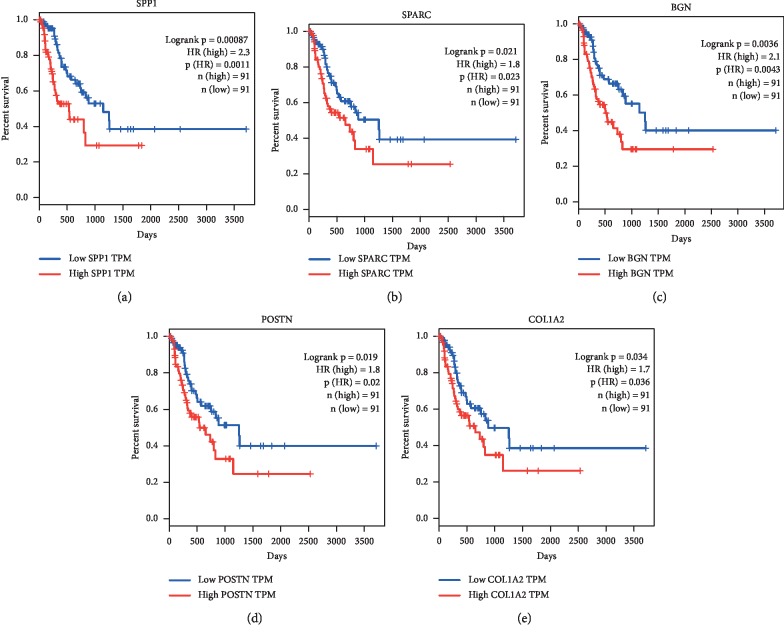
Prognostic value of five hub genes in ESCC obtained from GEPIA (http://gepia.cancer-pku.cn/index.html). High expression levels of SPP1 (a), SPARC (b), BGN (c), POSTN (d), and COL1A2 (e) were associated with poor disease-free survival.

The expression levels of the hub genes in the cancer tissues were significantly higher than those in healthy control tissues, except for *SNAI2*, according to the data from GEPIA (http://gepia.cancer-pku.cn/index.html) (Figure 5). DFS analysis of the hub genes demonstrated that high mRNA expression levels of SPP1 (HR: 2.3, *P*=0.00087), SPARC (HR: 1.8, *P*=0.021), BGN (HR: 2.1, *P*=0.0036), POSTN (HR: 1.8, *P*=0.019), and COL1A2 (HR: 1.7, *P*=0.034) were related to a poor prognosis in ESCC patients (Figure 6).

## 4. Discussion

In this study, 345 DEGs between ESCC and normal esophageal samples were identified from three microarray datasets in the GEO database, which were mainly significantly enriched in neutrophil-mediated immunity and cell cycle processes. In accordance with the GO annotation results, the KEGG pathway analysis of the DEGs and the two main clustering modules also suggested that a disordered cell cycle phase, unstable endocytosis, and unbalanced protein digestion and absorption affect the prognosis of patients with ESCC.

The cell cycle, a sequence of biological processes causing cell division and duplication, is crucial for the controlled proliferation and growth of the cell, and an unstable cell cycle process significantly impacts carcinogenesis and tumor progression, representing a key tumor characteristic [20–23]. In particular, the DEGs identified to be associated with ESCC in the present study were suggested to have an influence on chromosome segregation at the mitosis stage. Indeed, genes related to the cell cycle (*CDKN2A*, *RB1*, *NFE2L2*, *CHEK1*, and *CHEK2*) have been found to contain mutations in 2–10% of ESCC cases [24]. In addition to a role in the cell cycle, the overlapping DEGs in the three ESCC databases were related to neutrophil-associated processes. Neutrophils are the main defense cells that protect the body from microbial infection and eliminate pathogens [25]. Recent studies have revealed that neutrophil infiltration is closely related to the progression of different types of tumors. Given their plastic nature, neutrophils can differentiate into either a protumoral (N2) or an antitumoral (N1) phenotype depending on the tumor background, which play opposite roles in tumor development [26]. Chen et al. [27] showed that tumor-infiltrating MPO + neutrophils are a favorable prognostic factor for ESCC; however, there is still insufficient evidence to reveal the exact relationship between neutrophil activation and ESCC. In addition to the above, our results indicated that endocytosis, DNA replication, and pancreatic secretion signaling pathways were involved in the development of ESCC.

Among the DEGs, 21 hub genes were identified in the PPI network, and five of these genes, namely, *SPP1*, *SPARC*, *BGN*, *POSTN*, and *COL1A2*, were associated with the DFS of ESCC patients, in which higher expression levels of these genes were associated with a shorter DFS. SPP1 is a secreted glycoprotein that has been closely associated with the metastasis of various tumors such as gastric cancer, breast cancer, and melanoma [28–30]. Lin et al. [31] observed that all five subtypes of the *SPP1* gene are coexpressed in most primary EACs and could promote the invasion and dissemination of tumor cells. However, the clinical value of SPP1 in ESCC is rarely mentioned. Only one study found that serum levels of SPP1 in ESCC patients were higher than those in healthy controls [32]. SPARC is a matricellular protein that modulates cell adhesion and growth, along with cell-matrix interactions by binding to the extracellular matrix [33], and was suggested as a candidate biomarker for diagnosing invasive meningiomas [34]. Compared with the normal epithelium, the mRNA and protein expression levels of SPARC were found to be substantially higher in tumor tissues [35]. Moreover, overexpression of *SPARC* was correlated with a poor prognosis of patients with EAC [36]. Another study showed a significant difference in the *SPARC* levels in tumor tissue between gastric cancer and ESCC (15% vs. 34%) [37], suggesting SPARC as a potential novel therapeutic target. High expression levels of BGN have also been detected in a variety of human epithelial cancers [38, 39], indicating a potentially important role in tumor development. In addition, patients with high BGN levels were associated with significantly worse DFS than those with low expression, suggesting that higher expression of BGN indicates invasive tumor behavior and predicts poor clinical outcome in ESCC patients [40]. POSTN is a vital downstream target in the transforming growth factor-*β* signaling pathway, which plays an essential role in the process of triggering and promoting the epithelial-mesenchymal transition, a key step in the induction of malignant characteristics in cancer cells [41, 42]. Recent studies have suggested that overexpression of POSTN mediates the progression of EC [43]. POSTN was also found to be involved in the epithelial-mesenchymal transition of ESCC cells and was suggested as a predictive factor for tumor invasion and metastasis [44]. COL1A2 is a subtype of type I collagen, which is essential for maintaining the structure of interstitial spaces, along with the skin, gut, and breast. Wong et al. [45] discovered that *COL1A2* might serve as a biomarker in ESCC based on bioinformatics analysis. Therefore, the influence of COL1A2 on the pathogenesis and prognosis of ESCC warrants further experimental validation and exploration.

## 5. Conclusions

In summary, we identified 345 DEGs and 21 hub genes in ESCC from three gene profile datasets using an integrated bioinformatics approach. Among them, SPP1, SPARC, BGN, POSTN, and COL1A2 may emerge as potential prognostic biomarkers or therapeutic targets for ESCC. Functional annotations of the common DEGs in the three datasets indicate that cell cycle and neutrophil activation might be the main biological processes for the development and progression of ESCC and that DNA replication, endocytosis, and protein digestion and absorption signaling pathways also participate in the ESCC process. Although further experimental studies are required to verify these results, our data provide clues to guide the future exploration of prognostic biomarkers and molecular targeted therapy for ESCC.

## Figures and Tables

**Table 1:** Information of three GEO datasets in this study.

Datasets	Platform	Samples (tumor/normal)
GSE20347	Affymetrix human genome U133A 2.0 array	17/17
GSE23400	Affymetrix human genome U133A array	53/53
GSE26886	Affymetrix human genome U133 plus 2.0 array	19/9

**Table 2:** 71 genes were identified by 12 differential analysis methods, and 21 genes were detached by at least 5 methods.

Genes	Times	Genes	Times	Genes	Times	Genes	Times
MMP1	9	PLAU	5	MCM3	3	COL5A2	1
MMP9	9	SPARC	5	MCM7	3	COL6A3	1
COL3A1	8	VCAN	5	SCEL	3	CRABP2	1
CXCL8	8	CDKN3	4	THBS2	3	CRCT1	1
UBE2C	8	COL5A1	4	TTK	3	CXCL1	1
BGN	7	ISG15	4	ANO1	2	GABRP	1
MMP13	7	KRT4	4	CDH11	2	GMPS	1
POSTN	7	MCM2	4	CENPF	2	IFI44L	1
SPP1	7	MCM4	4	CKS1B	2	IFI6	1
ITGA6	6	MUC1	4	COL11A1	2	IRS1	1
LUM	6	ASPM	3	CXCR2	2	LCN2	1
RFC4	6	CKS2	3	MCM6	2	MMP12	1
SNAI2	6	DLGAP5	3	SLPI	2	MYH11	1
COL1A2	5	DTL	3	TYMS	2	PSCA	1
COL4A1	5	ERBB3	3	ADH7	1	PTHLH	1
ECT2	5	ESPL1	3	ALCAM	1	S100A9	1
IGFBP3	5	FANCI	3	ANXA1	1	SAMD9	1
MMP3	5	KIF14	3	CCNG2	1		

## Data Availability

The data used to support this study are from prior studies and datasets, which have been cited at relevant places within the text as references [8–10]. The processed data are included within the article and the supplementary information file

## Supplementary Materials

Supplementary Materials Table S1: A total of 345 overlapping DEGs were identified from GSE20347, GSE23400, and GSE26886.
